# Male non-insulin users with type 2 diabetes mellitus are predisposed to gastric corpus-predominant inflammation after *H. pylori* infection

**DOI:** 10.1186/s12929-017-0389-x

**Published:** 2017-10-30

**Authors:** Yao-Jong Yang, Chung-Tai Wu, Horng-Yih Ou, Chin-Han Lin, Hsiu-Chi Cheng, Wei-Lun Chang, Wei-Ying Chen, Hsiao-Bai Yang, Cheng-Chan Lu, Bor-Shyang Sheu

**Affiliations:** 1 Departments of Pediatrics, National Cheng Kung University Hospital, College of Medicine, National Cheng Kung University, Tainan, Taiwan; 2 Internal Medicine, National Cheng Kung University Hospital, College of Medicine, National Cheng Kung University, Tainan, Taiwan; 3 Pathology, National Cheng Kung University Hospital, College of Medicine, National Cheng Kung University, Tainan, Taiwan; 4 Institutes of Clinical Medicine, National Cheng Kung University Hospital, College of Medicine, National Cheng Kung University, Tainan, Taiwan; 5 Department of Pathology, Ton-Yen General Hospital, Hsin-Chu, County, Taiwan; 6 Department of Internal Medicine, Tainan Hospital, Ministry of Health and Welfare, Tainan, Taiwan; 7 Department of Internal Medicine, National Cheng Kung University Hospital, 138 Sheng Li Road, Tainan, 70428 Taiwan

**Keywords:** *H. Pylori*, Type 2 diabetes mellitus, Gastric cancer, Gender, Insulin

## Abstract

**Background:**

Both *H. pylori* infection and diabetes increase the risk of gastric cancer. This study investigated whether patients with type 2 diabetes mellitus (T2DM) and *H. pylori* infection had more severe corpus gastric inflammation and higher prevalence of precancerous lesions than non-diabetic controls.

**Methods:**

A total of 797 patients with type 2 diabetes mellitus were screened for *H. pylori*, of whom 264 had *H. pylori* infection. Of these patients, 129 received esophagogastroduodenoscopy to obtain topographic gastric specimens for gastric histology according to the modified Updated Sydney System, corpus-predominant gastritis index (CGI), Operative Link on Gastritis Assessment, and Operative Link on Gastric Intestinal Metaplasia Assessment. Non-diabetic dyspeptic patients who had *H. pylori* infection confirmed by esophagogastroduodenoscopy were enrolled as controls.

**Results:**

The male as well as total T2DM patients had higher acute/chronic inflammatory and lymphoid follicle scores in the corpus than non-diabetic controls (*p* < 0.05). In contrast, the female T2DM patients had higher chronic inflammatory scores in the antrum than the controls (*p* < 0.05). In T2DM patients, the males had significantly higher rates of CGI than the females (*p* < 0.05). Multivariate logistic regression analysis showed that male patients (odds ratio: 2.28, 95% confidence interval: 1.11–4.69, *p* = 0.025) and non-insulin users (odds ratio: 0.33, 95% confidence interval: 0.15–0.74, *p* = 0.007) were independent factors for the presence of CGI in the *H. pylori*-infected patients with type 2 diabetes mellitus.

**Conclusions:**

Patients with type 2 diabetes mellitus and *H. pylori* infection had more severe corpus gastric inflammation than non-diabetic controls. Moreover, male gender and non-insulin users of T2DM patients were predisposed to have corpus-predominant gastritis after *H. pylori* infection.

**Trial registration:**

ClinicalTrial: NCT02466919, retrospectively registered may 17, 2015.

## Background

*H. pylori* infection is known to increase the risk of gastric cancers [1, 2], and both chronic atrophic gastritis and intestinal metaplasia have been reported to be precancerous lesions that can potentially develop into gastric adenocarcinoma [3, 4]. The Operative Link on Gastritis Assessment (OLGA) and Operative Link on Gastric Intestinal Metaplasia Assessment (OLGIM) gastritis staging systems are based on the histology of both antrum and corpus tissues and can be applied to assess the risk of gastric cancer in patients with premalignant lesions [5, 6]. A more advanced OLGA and OLGIM stage indicates corpus predominance and higher risk of gastric cancer. Our recent previous study further disclosed that the corpus-predominant gastritis index (CGI) can be used to identify *H. pylori*-infected patients currently without intestinal metaplasia who are at an increased risk of gastric cancer earlier than the OLGA and OLGIM staging systems [7]. Accordingly, if the high-risk group can be identified earlier by gastric histology, *H. pylori* eradication can be performed to control the risk of gastric cancer [8].

Patients with diabetes mellitus (DM) and poor glycemic control are associated with a higher prevalence of gastrointestinal symptoms [9]. *H. pylori* infection has also been associated with insulin resistance and the development of type 2 diabetes mellitus (T2DM) [10–12]. Nevertheless, it remains controversial whether patients with diabetes are predisposed to *H. pylori* infection [13–16]. Several recent studies have shown that patients with DM who live in areas with a higher prevalence of *H. pylori* and gastric cancer and those with a longer duration of diabetes and poor glycemic control are predisposed to an increased risk of gastric cancer [17–19]. These observations suggest that even though the association between T2DM and a higher rate of *H. pylori* infection is controversial, the risk of gastric cancer is higher in patients with T2DM once infected with *H. pylori*. Therefore, the early detection and prompt treatment of *H. pylori* infection should be a priority to reduce the risk of gastric cancer in patients with T2DM.

Quatrinie et al. reported 23 diabetic patients with active gastritis, 25% of whom had intestinal metaplasia [14]. In contrast, Małlecki et al. reported a lower prevalence (41% vs. 71%) of chronic active gastritis in 39 patients with DM than in non-diabetic controls [20]. Therefore, we conducted this large-scale comparison between patients with T2DM and non-diabetic dyspeptic controls to investigate whether T2DM predisposes patients to a higher rate of precancerous lesions, more advanced OLGA or OLGIM stage, or a higher rate of CGI to predict the risk of gastric cancer after *H. pylori* infection. Our results disclosed that male patients and non-insulin users with T2DM were predisposed to have CGI. These findings may offer a promising risk factor stratification tool to identify the high-risk group of patients with T2DM who would benefit from earlier *H. pylori* eradication to control gastric cancer.

## Methods

### Patient characteristics, recruitment, and eligibility

**Fig. 1:**
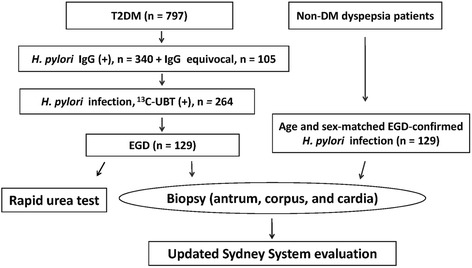
The schematic flow chart of the study protocol and case numbers

We enrolled patients aged ≥ 20 years diagnosed with T2DM who were regularly followed up at the out-patient clinic of National Cheng Kung University Hospital. The diagnostic criteria of T2DM were based on the criteria of American Diabetes Association [21]. The schematic flow chart of the study protocol is shown in Fig. 1 **.** The Ethical Committee of our institute approved the study design (A-BR-103-021). The patient’s sera were sent for anti-*H. pylori* IgG antibody testing (HEL-p TEST™ II; AMRAD Biotech, Perth, Western Australia) using an enzyme-linked immunosorbent assay (ELISA) after obtaining informed consent. Patients with seropositive and borderline seropositive results were further confirmed by ^13^C–UBT. The cutoff value of positive ^13^C–UBT was defined as an excess ^13^CO2 ⁄ ^12^CO2 ratio of more than 4.0‰ [22]. Patients were excluded if they were pregnant, had a critical condition, previously received either successful or unsuccessful *H. pylori* eradication therapy, bleeding tendency (anti-platelet or anti-coagulant user), or contraindications for esophagogastroduodenoscopy. Age- and sex-matched *H. pylori*-infected non-diabetic patients who had undergone esophagogastroduodenoscopy for upper gastrointestinal diseases in the same period were enrolled as controls. The *H. pylori* infection of non-diabetic patients was defined by a positive culture or both rapid urea test and histology showing positive.

### Demographic and laboratory data

Among T2DM patients, demographic data including age, sex, body weight and height, duration of diabetes, and the use of oral and intravenous hypoglycemic drugs were recorded. The laboratory data included fasting sugar and glycated hemoglobin A1c (HbA1c) at the most recent clinical visit before endoscopy were collected in patietns with T2DM as well.

### Esophagogastroduodenoscopy and gastric histology

The patients with positive ^13^C–UBT results were invited to receive an esophagogastroduodenoscopy examination. The gastric biopsy samples were collected for the rapid urea test (one sample) and histology, including from the antrum (two samples), corpus (two samples), and cardia (one sample) sampled topographically as our previous reports [5–7, 23]. The same pathologist who was blinded to whether or not the patient had diabetes evaluated the histology according to the modified Updated Sydney System [24]. Acute inflammation score (AIS, range 0–3), chronic inflammation score (CIS, range 1–3), lymphoid follicle formation (LF, range 0–8), *H. pylori* density (HPD, range 0–5), atrophic change (AT, range 0–3), and intestinal metaplasia (IM, range 0–3) were scored as in our previous report [24]. The OLGA and OLGIM grading systems and the presence of CGI were based on our previous reports [5–7]. *H. pylori* infection was diagnosed according to a positive ^13^C–UBT result combined with either positive histology or rapid urea test.

### Statistical analysis

Categorical data related to baseline characteristics were evaluated using Pearson’s χ2 test or Fisher’s exact test. Continuous data were analyzed using the independent *t* test. The histological scores of parameters were compared between the patients with and without T2DM using the Mann-Whitney U test. Linear-by-linear regression and multivariate logistic regression analyses were used as appropriate to investigate the independent risk factors for CGI in the patients with T2DM infected with *H. pylori.* All tests were two-tailed, and a *P* value less than 0.05 was considered to be statistically significant.

## Results

### Demographic and endoscopic features of the patients with diabetes and the non-diabetic controls

Of the 797 screened patients with T2DM, the seropositive prevalence of *H. pylori* infection was 43%. Of these 797 patients, 264 (249 seropositive and 15 equivocal) had positive ^13^C–UBT results, of whom 129 received esophagogastroduodenoscopy to obtain gastric biopsies for histological evaluation (Fig. 1).

Among these 129 patients (56 females and 73 males), there were no significant differences in mean age between the females and males (61.0 vs. 59.2 years, *p* > 0.05, Table 1). The mean duration of diabetes in these 129 cases was 11.1 years. Forty-three (33.3%) of the patients had dyspepsia, nausea, and bloating. The endoscopic diagnoses included 74 (57.4%) cases with non-ulcer gastritis and 55 (23.6%) with peptic ulcers. We also enrolled 129 age- and sex-matched dyspeptic patients who had *H. pylori* infection confirmed by esophagogastroduodenoscopy (culture or rapid urea test/histology) during the same period as non-diabetic controls. The rate of non-ulcer gastritis was significantly lower in the controls than in the T2DM patients (45.0% vs. 57.4%, *p* < 0.05).

**Table 1:** Comparison of gastric histology assessed according to Updated Sydney System scores among patients with diabetes and non-diabetes controls stratified by gender

Characters/mean(SD) or %(n)		DM (*n* = 129)			Non-DM (n = 129)			*P* value DM vs. non-DM
		Male (*n* = 73)	Female (*n* = 56)	*P* value male vs. female	Male (n = 73)	Female (n = 56)	*P* value male vs. female	
Age (years)		59.2 (11.0)	61.0 (10.2)	0.35	59.4 (11.2)	60.8 (10.3)	0.46	–
Non-ulcers gastritis^a^		53.4 (39)	62.5 (35)	0.30	26.0 (19)	69.6 (39)	< 0.001	0.046
CIS^c^	Antrum^b^	2.85 (0.49)	2.95 (0.30)	0.18	2.81 (0.57)	2.77 (0.60)	0.60	0.13
	Body^a^	2.68 (0.62)	2.66 (0.72)	0.79	2.32 (0.85)	2.70 (0.57)	0.01	0.02
	Cardia	2.40 (0.81)	2.30 (0.87)	0.58	2.12 (0.91)	2.46 (0.79)	0.03	0.45
AIS^c^	Antrum	1.84 (0.62)	2.02 (0.40)	0.08	1.71 (0.81)	1.77 (0.85)	0.58	0.13
	Body^a^	1.40 (0.95)	1.54 (0.89)	0.42	1.05 (1.03)	1.39 (0.95)	0.06	0.04
	Cardia	1.16 (1.00)	0.86 (1.02)	0.09	0.90 (1.00)	1.16 (0.99	0.15	0.90
LF^c^	Antrum	2.15 (2.11)	2.32 (2.01)	0.52	1.64 (1.87)	1.84 (1.87)	0.55	0.04
	Body^a^	1.49 (1.81)	1.29 (1.72)	0.48	0.66 (1.11)	0.70 (1.04)	0.70	0.001
	Cardia	0.85 (1.53)	0.48 (0.89)	0.18	0.53 (0.96	0.48 (0.76)	0.96	0.74
HPD^c^	Antrum	3.36 (1.31)	3.39 (1.30)	0.92	3.29 (1.64)	3.23 (1.41)	0.60	0.94
	Body	3.21 (1.24)	3.36 (1.18)	0.42	3.16 (1.38)	3.41 (1.32)	0.32	0.77
	Cardia	2.75 (1.38)	2.63 (1.27)	0.61	2.77 (1.51)	2.98 (1.42	0.46	0.24
IM	Antrum	30.1 (22)	26.8 (15)	0.68	38.4 (28)	33.9 (18)	0.46	0.23
	Body	15.1 (11)	17.9 (10)	0.67	15.1 (11)	10.7 (6)	0.47	0.48
	Cardia	1.4 (1)	3.6 (2)	0.58	0	1.8 (1)	0.43	0.31
AT	Antrum	67.1 (49)	67.9 (38)	0.93	68.5 (50)	62.5 (35)	0.48	0.79
	Body	34.2 (25)	23.2 (13)	0.17	21.9 (16)	28.6 (16)	0.39	0.40
	Cardia	17.8 (13)	14.3 (8)	0.62	12.3 (9)	8.9 (5)	0.54	0.19

*SD* standard deviation, *CIS* chronic inflammation score, *AIS* acute inflammation score

*LF* lymphoid follicle, *HPD* H. pylori density, *IM* intestinal metaplasia, *AT* atrophy

*DM* Patients with diabetes, *Non-DM* patients without diabetes (controls)

^a^: *p* < 0.05, male patients with and without DM

^b^: p < 0.05, female patients with and without DM

^c^: Mann-Whitney U test

### Difference in gastric histology between the *H. pylori*-infected patients with T2DM and controls

The patients with T2DM had a significantly higher chronic inflammation score (2.67 vs. 2.48, *p* = 0.02), acute inflammatory score (1.46 vs. 1.20, *p* = 0.04), and lymphoid follicle formation score (1.40 vs. 0.67, *p* = 0.001) in the corpus compared to the controls. The lymphoid follicle formation score in the antrum was also higher in the patients than in the controls (2.22 vs. 1.73, *p* = 0.04). However, there were no significant differences in *H. pylori* density and the rates of intestinal metaplasia or atrophic change between the patients and controls in any topographic location (antrum, corpus, and cardia) (all *p* > 0.05).

Table 1 shows comparisons of topographic gastric histological features related to *H. pylori* infection between the patients with T2DM and controls stratified by gender. Only the male patients with T2DM but not the females gender had statistically significantly higher chronic inflammation score (2.68 vs. 2.32, *p* = 0.005), acute inflammatory score (1.40 vs. 1.05, *p* = 0.04), and lymphoid follicle formation score (1.49 vs. 0.66, *p* = 0.004) in the corpus than the controls. In contrast, the female patients with T2DM had a significantly higher chronic inflammation score in the antrum than the non-diabetic controls (2.95 vs. 2.77, *p* = 0.047). Moreover, there were no significant differences between genders in the rates of intestinal metaplasia and atrophic change between the diabetic patients and controls (all *p* > 0.05).

### Male patients with T2DM had a higher rate of CGI than female patients after *H. pylori* infection

Because the male patients with T2DM had more evident corpus inflammation than the male controls, we further validated whether the male patients with T2DM had a higher rate of CGI or more advanced stages of OLGA or OLGIM than the female patients or controls (Table 2). The male patients with T2DM had a higher rate of CGI than the female patients with T2DM (58.9%. vs. 39.2%, *p* = 0.03), but a similar rate of CGI to the non-diabetic male control (58.9% vs. 62.5%, *p* > 0.05). In addition, for both genders, there were no significant differences in the rate of either OLGA stage II or higher or OLGIM stage II or higher between the patients and controls (all *p* > 0.05; Table 2).

**Table 2:** The presence of CGI and OLGA and OLGIM grade stratified by gender between the patients with and without diabetes mellitus and non-diabetic controls

Gastritis staging % (n)	DM (n = 129)			Non-DM (n = 129)			*p* value DM vs. Non-DM
	Male (n = 73)	Female (n = 56)	*p* value OR (95% CI)	Male (n = 73)	Female (n = 56)	*p* value OR (95% CI)	
CGI^b^	58.9 (43)	39.3 (22)	0.03 2.2 (1.09–4.51)	52.1 (38)	62.5 (35)	0.24 0.6 (0.32–1.32)	0.32
OLGA ≥ II	54.8 (40)	50.0 (28)	0.59 1.2 (0.60–2.44)	43.8 (32)	50.0 (28)	0.49 0.8 (0.39–1.57)	0.32
OLGIM ≥ II	32.9 (24)	32.1 (18)	0.93 1.0 (0.49–2.18)	41.1 (30)	33.9 (19)	0.41 1.4 (0.66–2.80)	0.36

*DM* diabetes mellitus, *OR* odds ratio, *CI* confident interval

^b^: *p* < 0.05, female patients with and without DM

### Non-insulin users and male sex were risk factors for CGI in the *H. pylori*-infected patients with T2DM

**Fig. 2:**
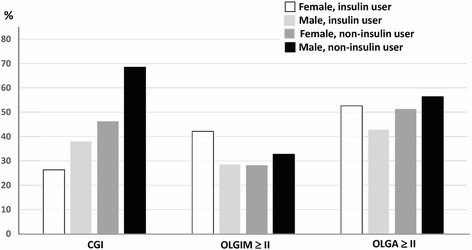
The ratio of precancerous lesions (CGI, OLGIM≥ II, OLGA≥ II) in 73 male and 56 female diabetic patients who did and did not use insulin. The trend of the risk of precancerous lesions was analyzed by linear-to-linear regression (CGI, *P* < 0.001; OLGIM≥ II, *P* = 0.64; OLGA≥ II, *P* = 0.51)

We further investigated whether any factors were associated with the presence of CGI in the 129 *H. pylori*-infected patients with T2DM (Table 3). The patients with T2DM with CGI were predominantly male (66.2% vs. 46.9%, *p* = 0.027) and fewer used insulin for glycemic control (18.5% vs. 42.2%, *p* = 0.003) than those without CGI. However, there were no significant differences in the mean age and duration of DM, rate of non-ulcer gastritis, body mass index, and the levels of fasting sugar and HbA1c between the patients with T2DM with and without CGI (*p* > 0.05). Multivariate logistic regression analysis revealed that male gender (odds ratio: 2.28, 95% confidence interval: 1.11–4.69, *p* = 0.025) and not using insulin (odds ratio: 0.33, 95% confidence interval: 0.15–0.74, *p* = 0.007) were independent risk factors for the presence of CGI in the *H. pylori*-infected patients with T2DM. The rates of CGI showed an upward trend for female insulin users, male insulin users, female non-insulin users, and male non-insulin users (*p* < 0.001 by linear-to-linear regression analysis, Fig. 2).

**Table 3:** The factors related to the presence of corpus-predominant gastritis index in 129 T2DM patients with *H. pylori* infection

Characteristic % (n) or mean (SD)	CGI		OR (95% CI)	*p* value
	Presence (*n* = 65)	Absence (*n* = 64)		
Female: Male	22: 43	34: 30	2.2 (1.09–4.51)	0.027
Insulin use	18.5 (12)	42.2 (27)	0.3 (0.14–0.69)	0.003
Age (years)	61.2 (8.0)	58.8 (12.8)		0.195
Body mass index	27.1 (5.6)	25.5 (3.9)		0.073
DM duration (year)	10.6 (7.6)	11.6 (8.2)		0.481
Fasting sugar (mg/dl)	141.1 (38.5)	142.6 (48.4)		0.858
HbA1c (%)	7.7 (1.3)	7.7 (1.6)		0.994
HbA1c ≤ 7%	32.3 (21)	40.6 (26)	0.7 (0.33–1.40)	0.293

*CGI* corpus-predominant gastritis index, *HbA1c* glycated hemoglobin A1c

*OR* odds ratio, *CI* confidence interval, *SD* standard deviation

## Discussions

The results of this large-scale, case-control study are highly original to illustrate that male patients with T2DM had higher chronic and acute inflammatory scores in the corpus than non-diabetic dyspeptic controls. Moreover, *H. pylori*-infected diabetic patients who were male and non-insulin users had a higher risk of CGI. This suggests that this high-risk group should receive earlier anti-*H. pylori* therapy to reduce the risk of gastric cancer.

*H. pylori* infection is defined as a type I World Health Organization carcinogen for gastric adenocarcinoma [25]. A large-scale meta-analysis showed that patients with diabetes had an increased risk of gastric cancer unrelated to their geographical region of residence. Moreover, after adjusting for the prevalence of *H. pylori* infection, the risk was two-fold higher [26]. These results suggest that diabetic patients with *H. pylori* infection are at a higher risk of gastric cancer, although the underlying mechanism remains unclear.

Chronic gastric inflammation has been reported to play a major role in gastric carcinogenesis [27, 28]. In addition, more severe chronic gastric inflammation in the corpus has been reported to result in an increased risk of gastric cancer [28]. We found that the patients with T2DM and *H. pylori* infection had more severe inflammation in the corpus than those without diabetes. Moreover, the aggregation of lymphoid hyperplasia, which indicates the severity and activity of mucosal inflammation [29], was also severe in both the antrum and corpus of the patients with T2DM. A greater severity of chronic gastric inflammation in the corpus in patients with T2DM may therefore lead to an increased risk of gastric cancer.

Males are known to have a higher risk of gastric cancer, and is similar in populations with high and low incidence of gastric cancer [30]. 17β-estradiol (E2) has been shown to reduce female gastric neutrophil infiltration and prevent *H. pylori*-induced gastric cancer in mice [31]. Other reproductive factors such as years of fertility and parity are associated to gastric cancer development [32, 33], however, the results are not consistent [34]. However, the accurately biological mechanism of gender difference in gastric cancer risk needs more detail study. In the current study, we found that *H. pylori*-infected male patients with T2DM but not female patients had higher levels of neutrophils and lymphocytes over the corpus (Table 1) compared to the controls. Moreover, the male patients with T2DM had a significantly higher rate of CGI than the female patients with T2DM (Table 2). These findings are consistent with the general concept that males are at a higher risk of gastric cancer, and also that male patients with T2DM are at a higher risk of gastric precancerous lesions than females.

Insulin is the principle therapy for both patients with type 1 DM and also for those with T2DM with a poor response to oral hypoglycemic agents. We found that not using insulin was a significant factor for the development of CGI. Kim et al. reported that a lower fasting glucose level (<86 mg/dL) was significantly associated with an increased risk of distal gastric cancer compared to a normal level [35]. However, there were no significant differences in fasting sugar or HbA1c levels between the patients with and without CGI in this study. Furthermore, the insulin-like growth factor pathway has been reported to play a key role in the malignant progression of esophageal cancer in in vitro and in vivo studies [36]. In addition, a large follow-up cohort study showed that the use of insulin and glinides was associated with a higher risk of stomach cancer [37]. In contrast to our findings, a large population-based study reported that the use of insulin was not associated with the risk of gastric cancer [38]. Therefore, further large-scale longitudinal observational studies are needed to elucidate the association between the use of insulin in patients with diabetes and gastric malignancy.

Atrophic gastritis and intestinal metaplasia are known to be precancerous lesions predisposing to gastric carcinogenesis [3, 7]. Our previous study showed that patients with gastric cancer had a higher prevalence of CGI and OLGIM stage II-IV. In addition, CGI has been reported to be highly correlated to the presence of spasmolytic polypeptide-expressing metaplasia [7]. Although we did not find a higher grade of OLGA or OLGIM in the patients with T2DM compared to the controls, the male patients with T2DM had a higher rate of CGI than the female patients with T2DM (Table 3). This suggests that the higher prevalence of gastric cancer in *H. pylori*-infected male patients with T2DM may be caused by chronic corpus inflammation and CGI rather than intestinal metaplasia.

There are some limitations to this study. First, all of the non-diabetic control patients were dyspeptic, so that we could not investigate the role of dyspeptic symptoms in the development of gastric precancerous lesions. Furthermore, the other risk factors related to gastric cancer such as cigarette smoking, dietary habits, and body mass index were not available in controls. Second, the analysis of hypoglycemic drugs for precancerous lesions was limited to insulin. Third, the exact mechanism by which chronic inflammation rather than chronic atrophic gastritis-intestinal metaplasia induces gastric carcinogenesis in patients with T2DM remains unclear.

## Conclusions

In conclusion, the patients with T2DM had more severe corpus gastric inflammation than the non-diabetic controls after *H. pylori* infection. Male sex and not using insulin were independent risk factors for CGI after *H. pylori* infection in the patients with T2DM. Treating male patients with T2DM who do not use insulin for *H. pylori* treatment may be promising in reducing the risk of gastric cancer.

## Abbreviations

^13^C–UBT: ^13^C–Urea breath test
CGI: Corpus-predominant gastritis index
OLGA: Operative link on gastritis assessment
OLGIM: Operative link on gastric intestinal metaplasia assessment
T2DM: Type 2 diabetes mellitus

## References

[1] Uemura N, Okamoto S, Yamamoto S, Matsumura N, Yamaguchi S, Yamakido M (2001). *Helicobacter pylori* infection and the development of gastric cancer. N Engl J Med.

[2] Wroblewski LE, Peek RM, Wilson KT (2010). *Helicobacter pylori* and gastric cancer: factors that modulate disease risk. Clin Microbiol Rev.

[3] Zhang RG, Duan GC, Fan QT, Chen SY (2016). Role of *Helicobacter pylori* infection in pathogenesis of gastric carcinoma. World J Gastrointest Pathophysiol.

[4] Watari J, Chen N, Amenta PS, Fukui H, Oshima T, Tomita T (2014). *Helicobacter pylori* associated chronic gastritis, clinical syndromes, precancerous lesions, and pathogenesis of gastric cancer development. World J Gastroenterol.

[5] Rugge M, Meggio A, Pennelli G, Piscioli F, Giacomelli L, De Pretis G, et al. Gastritis staging in clinical practice: the OLGA staging system. Gut. 2007;56(5):631−636. 10.1136/gut.2006.106666 PMC1942143 17142647

[6] Capelle LG, de Vries AC, Haringsma J, Ter Borg F, de Vries RA, Bruno MJ (2010). The staging of gastritis with the OLGA system by using intestinal metaplasia as an accurate alternative for atrophic gastritis. Gastrointest Endosc.

[7] Tsai YC, Hsiao WH, Yang HB, Cheng HC, Chang WL, Lu CC (2013). The corpus-predominant gastritis index may serve as an early marker of *Helicobacter pylori*-infected patients at risk of gastric cancer. Aliment Pharmacol Ther.

[8] Malfertheiner P, Megraud F, O'Morain CA, Atherton J, Axon AT, Bazzoli F (2012). Management of *Helicobacter pylori* infection--the Maastricht IV/ Florence consensus report. Gut.

[9] Bytzer P, Talley NJ, Leemon M, Young LJ, Jones MP, Horowitz M (2001). Prevalence of gastrointestinal symptoms associated with diabetes mellitus: a population-based survey of 15,000 adults. Arch Intern Med.

[10] Gunji T, Matsuhashi N, Sato H, Fujibayashi K, Okumura M, Sasabe N, et al. *Helicobacter pylori* infection significantly increases insulin resistance in the asymptomatic Japanese population. Helicobacter. 2009;14(5):144−150. 10.1111/j.1523-5378.2009.00705.x 19751440

[11] Chen Y, Blaser MJ (2012). Association between gastric *Helicobacter pylori* colonization and glycated hemoglobin levels. J Infect Dis.

[12] Hsieh MC, Wang SS, Hsieh YT, Kuo FC, Soon MS, Wu DC (2013). *Helicobacter pylori* infection associated with high HbA1c and type 2 diabetes. Eur J Clin Investig.

[13] Oldenburg B, Diepersloot RJ, Hoekstra JB (1996). High seroprevalence of *Helicobacter pylori* in diabetes mellitus patients. Dig Dis Sci.

[14] Quatrini M, Boarino V, Ghidoni A, Baldassarri AR, Bianchi PA, Bardella MT (2001). *Helicobacter pylori* prevalence in patients with diabetes and its relationship to dyspeptic symptoms. J Clin Gastroenterol.

[15] Oluyemi A, Anomneze E, Smith S, Fasanmade O (2012). Prevalence of a marker of active *Helicobacter pylori* infection among patients with type 2 diabetes mellitus in Lagos, Nigeria. BMC Res Notes.

[16] Demir M, Gokturk HS, Ozturk NA, Kulaksizoglu M, Serin E, Yilmaz U (2008). *Helicobacter pylori* prevalence in diabetes mellitus patients with dyspeptic symptoms and its relationship to glycemic control and late complications. Dig Dis Sci.

[17] Chen YL, Cheng KC, Lai SW, Tsai IJ, Lin CC, Sung FC (2013). Diabetes and risk of subsequent gastric cancer: a population-based cohort study in Taiwan. Gastric Cancer.

[18] Hidaka A, Sasazuki S, Goto A, Sawada N, Shimazu T, Yamaji T (2015). Plasma insulin, C-peptide and blood glucose and the risk of gastric cancer: the Japan public health center-based prospective study. Int J Cancer.

[19] Marimuthu SP, Vijayaragavan P, Moysich KB, Jayaprakash V (2011). Diabetes mellitus and gastric carcinoma: is there an association?. J Carcinog.

[20] Małlecki M, Bień AI, Galicka-Latałla D, Stachura J, Sieradzki J (1996). The prevalence of *Helicobacter pylori* infection and types of gastritis in diabetic patients. The Kraków study. Exp Clin Endocrinol Diabetes.

[21] American Diabetes Association (2010). Diagnosis and classification of diabetes mellitus. Diabetes Care.

[22] Yang YJ, Sheu BS, Lee SC, Yang HB, Wu JJ (2005). Children of *H. pylori*-infected dyspeptic mothers are predisposed to *H. pylori* acquisition with subsequent iron deficiency and growth retardation. Helicobacter.

[23] Yang YJ, JJ W, Sheu BS, Chen CR, CC L, Yang HBH (2008). *pylori* infection can change the intensity of gastric Lewis antigen expressions differently between adults and children. J Biomed Sci.

[24] Dixon MF, Genta RM, Yardley JH, Correa P (1996). Classification and grading of gastritis: the updated Sydney system: international workshop on the histology of gastritis, Houston 1994. Am J Surg Pathol.

[25] International Agency for Research on Cancer (1994). IARC monographs on the evaluation of carcinogenic risk to humans, vol 61: Schistosomotes, liver flukes and *Helicobacter pylori*.

[26] Yoon JM, Son KY, Eom CS, Durrance D, Park SM (2013). Pre-existing diabetes mellitusincreases the risk of gastric cancer: a meta-analysis. World J Gastroenterol.

[27] Balkwill F, Mantovani A (2001). Inflammation and cancer: back to Virchow?. Lancet.

[28] Fu H, Ma Y, Yang M, Zhang C, Huang H, Xia Y (2016). Persisting and increasing neutrophil infiltration associates with gastric carcinogenesis and E-cadherin downregulation. Sci Rep.

[29] Chen XY, Liu WZ, Shi Y, Zhang DZ, Xiao SD, Tytgat GN (2002). *Helicobacter pylori* associated gastric diseases and lymphoid tissue hyperplasia in gastric antral mucosa. J Clin Pathol.

[30] Sipponen P, Correa P (2002). Delayed rise in incidence of gastric cancer in females results in unique sex ratio (M/F) pattern: etiologic hypothesis. Gastric Cancer.

[31] Sheh A, Ge Z, Parry NM, Muthupalani S, Rager JE, Raczynski AR (2011). 17β-estradiol and tamoxifen prevent gastric cancer by modulating leukocyte recruitment and oncogenic pathways in *Helicobacter pylori*-infected INS-GAS male mice. Cancer Prev Res (Phila).

[32] Freedman ND, Chow WH, Gao YT, Shu XO, Ji BT, Yang G (2007). Menstrual and reproductive factors and gastric cancer risk in a large prospective study of women. Gut.

[33] Plesko I, Preston-Martin S, Day NE, Tzonou A, Dimitrova E, Somogyi J (1985). Parity and cancer risk in Slovakia. Int J Cancer.

[34] Inoue M, Ito LS, Tajima K, Yamamura Y, Kodera Y, Takezaki T (2002). Height, weight, menstrual and reproductive factors and risk of gastric cancer among Japanese postmenopausal women: analysis by subsite and histologic subtype. Int J Cancer.

[35] Kim TJ, Lee H, Min YW, Min BH, Lee JH, Son HJ (2016). Diabetic biomarkers and the risk of proximal or distal gastric cancer. J Gastroenterol Hepatol.

[36] Doyle SL, Donohoe CL, Finn SP, Howard JM, Lithander FE, Reynolds JV (2012). IGF-1 and its receptor in esophageal cancer: association with adenocarcinoma and visceral obesity. Am J Gastroenterol.

[37] Chang CH, Lin JW, Wu LC, Lai MS, Chuang LM (2012). Oral insulin secretagogues, insulin, and cancer risk in type 2 diabetes mellitus. J Clin Endocrinol Metab.

[38] Tseng CH (2013). Diabetes, insulin use, and gastric cancer: a population-based analysis of the Taiwanese. J Clin Gastroenterol.

